# Stenotrophomonas Necrotizing Tonsilitis in a Pediatric Patient: A Rare Presentation With Upper Airway Obstruction

**DOI:** 10.7759/cureus.69627

**Published:** 2024-09-18

**Authors:** Ting Jie Oui, Bee See Goh, Farah Liana Lokman, Thean Yean Kew, Rufinah Teo

**Affiliations:** 1 Otorhinolaryngology - Head and Neck Surgery, Universiti Kebangsaan Malaysia Medical Centre, Kuala Lumpur, MYS; 2 Radiology, Universiti Kebangsaan Malaysia Medical Centre, Kuala Lumpur, MYS; 3 Anesthesiology and Intensive Care, Universiti Kebangsaan Malaysia Medical Centre, Kuala Lumpur, MYS

**Keywords:** community-acquired, necrotizing tonsilitis, paediatric, peritonsillar abscess, retropharyngeal abscess, sternotrophomonas maltophilia

## Abstract

*Stenotrophomonas maltophilia* is notorious for its intrinsic resistance to many commonly used antibiotics, making it a particularly challenging pathogen to treat. It often causes severe opportunistic infections in immunocompromised and hospitalized patients. The potential for this infection to become fulminant with high mortality rates in both adults and children necessitates a multidisciplinary approach. Prompt initiation of appropriate antibiotic therapy can be lifesaving. In this case report, we present a rare instance involving an eight-month-old previously healthy infant diagnosed with necrotizing tonsillitis and a retropharyngeal abscess, which led to upper airway obstruction. Intraoperative tissue cultures identified this highly virulent gram-negative bacillus. The report details the medical and surgical management that resulted in the patient’s complete recovery.

## Introduction

*Stenotrophomonas maltophilia* is an aerobic, gram-negative bacillus known for its ability to form biofilms and its multidrug-resistant profile, which allows it to withstand many commonly used first-line, broad-spectrum antibiotics. This pathogen can cause severe opportunistic infections with mortality rates ranging from 6% to 40% [1]. It is typically transmitted via direct contact and is often found in hospitalized and immunocompromised patients. Children who have been hospitalized for extended periods and exposed to broad-spectrum antibiotics are particularly vulnerable to bacteremia caused by this resistant microorganism. To the best of our knowledge, this is the first reported case of necrotizing tonsillitis secondary to an* S. maltophilia *superinfection in a pediatric patient.

## Case presentation

We present a case report of a healthy, fully immunized eight-month-old boy diagnosed with community-acquired necrotizing tonsillitis complicated by extensive deep-neck abscesses. He initially presented with a high-grade fever lasting five days, vomiting, reduced oral intake, and lethargy. He was first seen at a private clinic, where he was treated for acute tonsillitis. At that time, there were no difficulties with swallowing or signs of neck swelling, and no symptoms suggestive of impending upper airway obstruction, such as noisy breathing or shortness of breath. The child’s antenatal period and birth history were uneventful, and there were no identifiable sick contacts. Despite more than 72 hours of oral antibiotics, his condition failed to improve, prompting a visit to the ED on the fifth day of illness.

On physical examination, the child was febrile and lethargic, but his hydration status was adequate, with a capillary refill time of less than two seconds. He was mildly tachycardic and had a high-grade fever of 39.5 °C. There were no signs of respiratory distress. Oropharyngeal examination revealed an inflamed posterior pharyngeal wall and grade 2 tonsils without overlying exudates. Systemic examinations were otherwise unremarkable, except for poor weight gain; he weighed only 5.7 kg, which was -2 standard deviations on the WHO Child Growth Standards chart. Initial blood investigations showed a hemoglobin level of 10.1 g/dL, a normal total white cell count of 4.24 × 10^9^/L, and a markedly elevated CRP level of 101 mg/dL. Other parameters were normal. The patient was admitted for close monitoring, IV maintenance fluids, and initiation of IV C-penicillin QID (50,000 IU/kg/dose).

Despite receiving IV antibiotics, the patient’s condition deteriorated. He became increasingly irritable, developed dysphagia with worsening oral intake, and exhibited neck stiffness. He also experienced recurrent temperature spikes and rigors. Examination revealed the child to be restless, lethargic, and showing signs of respiratory distress, including nasal flaring, tachypnea, and subcostal and intercostal recessions. His neck was hyperextended, stridor was present, and he was significantly tachycardiac. Oropharyngeal examination showed bilateral tonsillar exudates but no grayish membrane. There were multiple bilateral cervical lymphadenopathies. Due to worsening respiratory distress, the patient required transfer to the pediatric intensive care unit (PICU), where he was placed on continuous positive airway pressure support and subsequently intubated for airway protection. Antibiotics were escalated to IV ceftriaxone BD (50 mg/kg/dose) and IV metronidazole TDS (7.5 mg/kg/dose).

**Figure 1:**
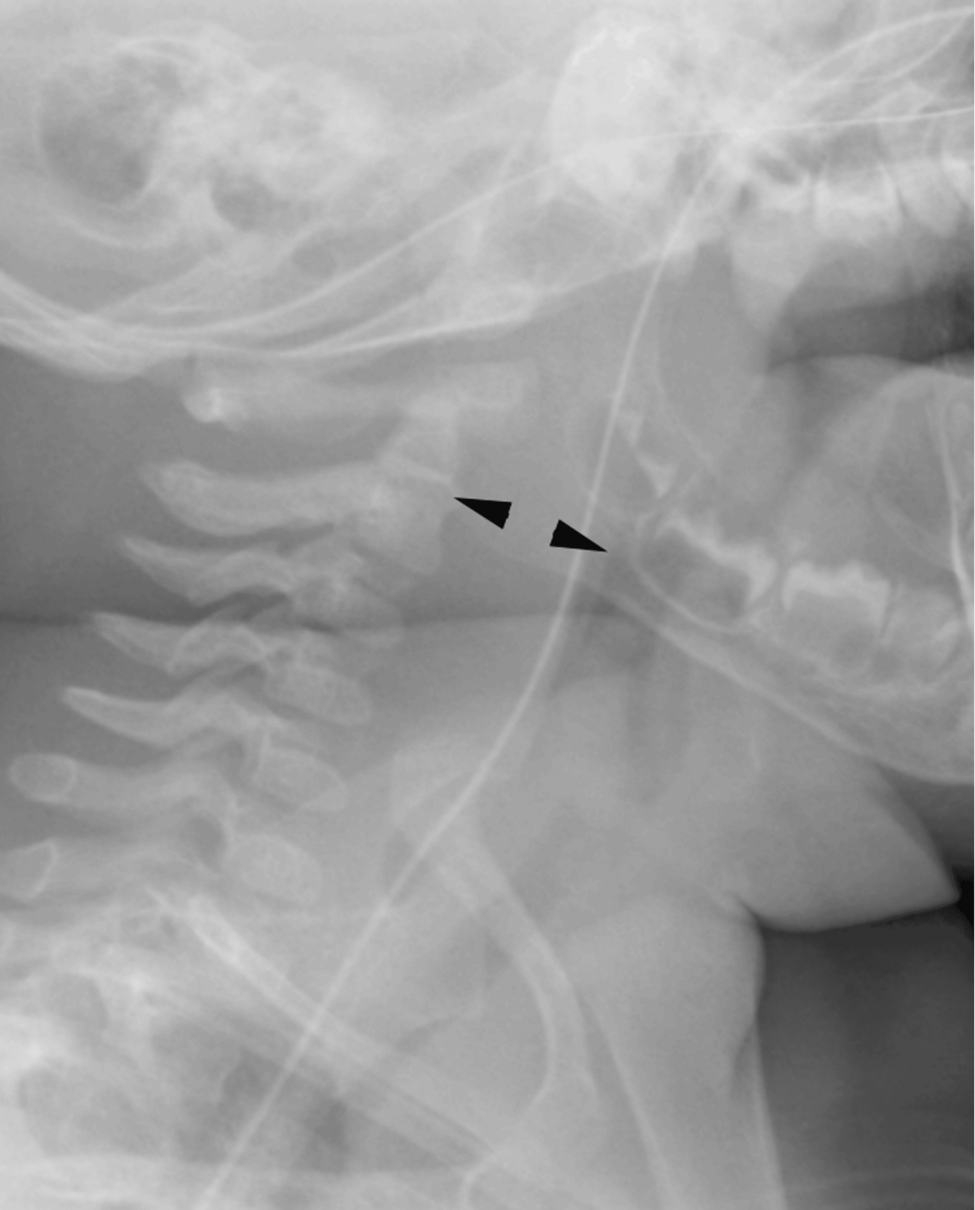
Lateral neck radiograph showing widening of the prevertebral space (arrowheads), measuring up to 13 mm at the C2 level and 14 mm at the C6 level. No gas shadows were observed. The airway was displaced anteriorly but without significant stenosis. No hyperdense foreign body or suspicious lytic cervical spinal lesions were detected.

**Figure 2:**
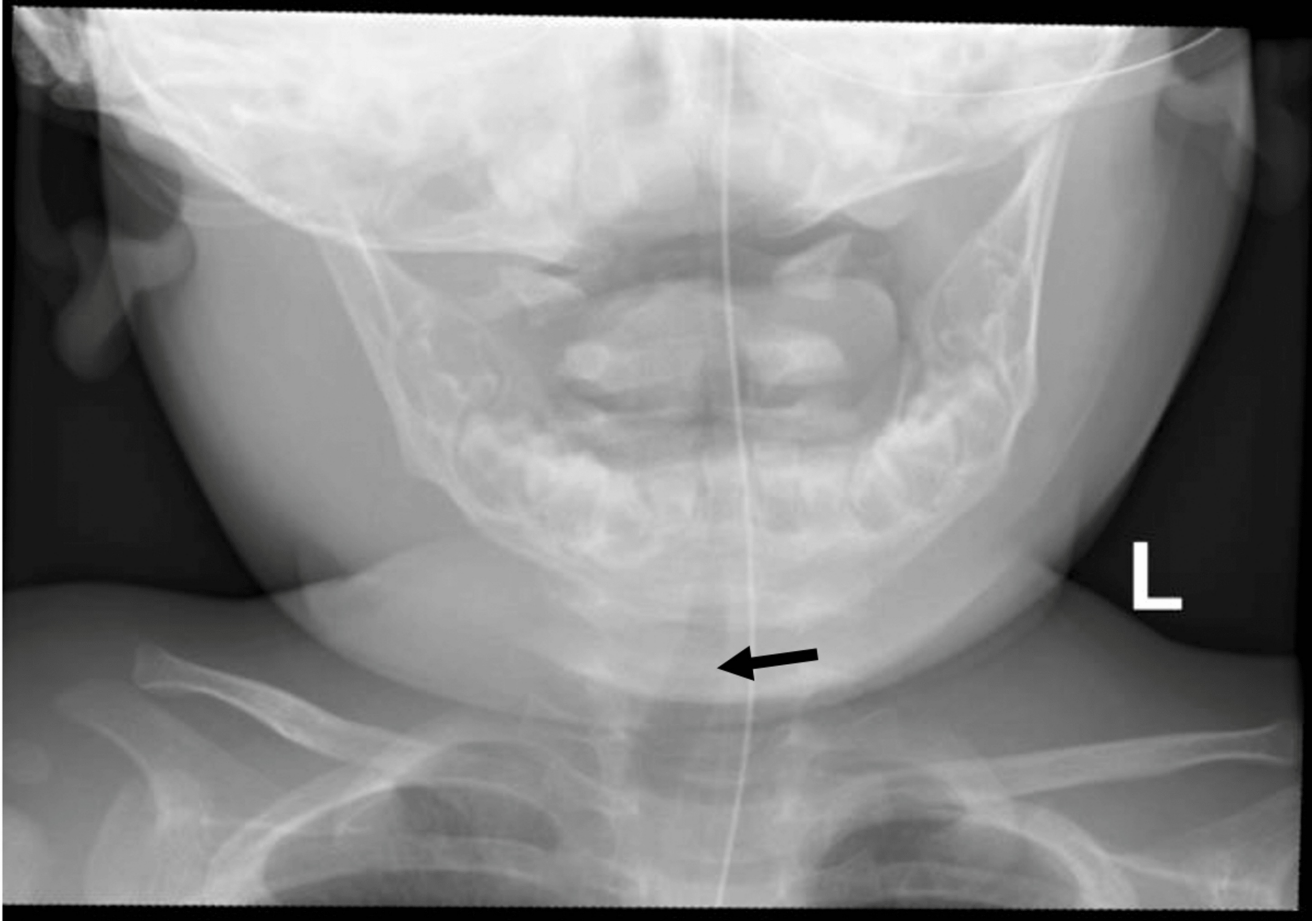
AP cervical radiograph showing the trachea (black arrow) displaced to the right. No significant narrowing is noted.

Lateral soft-tissue neck radiographs revealed significant prevertebral soft tissue thickening of 14 mm at both the C2 and C6 vertebrae (Figure 1). AP cervical radiographs showed narrowing of the upper airway with slight tracheal deviation to the right side (Figure 2). These radiographic findings suggested the presence of a retropharyngeal collection.

**Figure 3:**
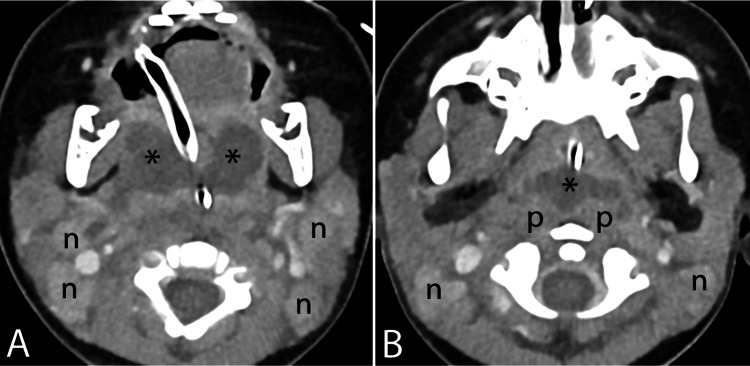
First CECT of the child showing axial planes at the level of the palatine tonsils (A) and slightly more superiorly at the pterygoid plates (B), with endotracheal and nasogastric catheters in place. Bilateral paired collections with rim enhancement were observed in the tonsillar fossae (asterisks in A), positioned directly lateral to the endotracheal catheter, replacing the normal soft tissue density of the palatine tonsils. The anterior extent of the collection reached the base of the tongue, while inferiorly it extended to the valleculae, just superior to the hyoid bone. Superiorly, adenoid hypertrophy was noted (not shown). Posterolaterally, fluid hypodensity was observed extending into the carotid spaces without rim enhancement. Posteriorly, part of the collection extended into the retropharyngeal space (asterisk in B), adjacent to the prevertebral longus coli muscles (marked as p), which appeared edematous but not infiltrated. There was no extension of the retropharyngeal collection inferiorly into the oropharynx or superior mediastinum. The palatine and lingual tonsils were not well delineated. Multiple enlarged enhancing cervical lymph nodes were present bilaterally at levels IIa and IIb (marked as n). CECT, contrast-enhanced CT

He then underwent a contrast-enhanced CT (CECT) scan of the neck and thorax. The scan revealed radiographic features consistent with bilateral peritonsillar abscesses extending into the vallecula, with surrounding extensive phlegmon and involvement of the retropharyngeal and prevertebral spaces (Figure 3A, 3B).

**Figure 4:**
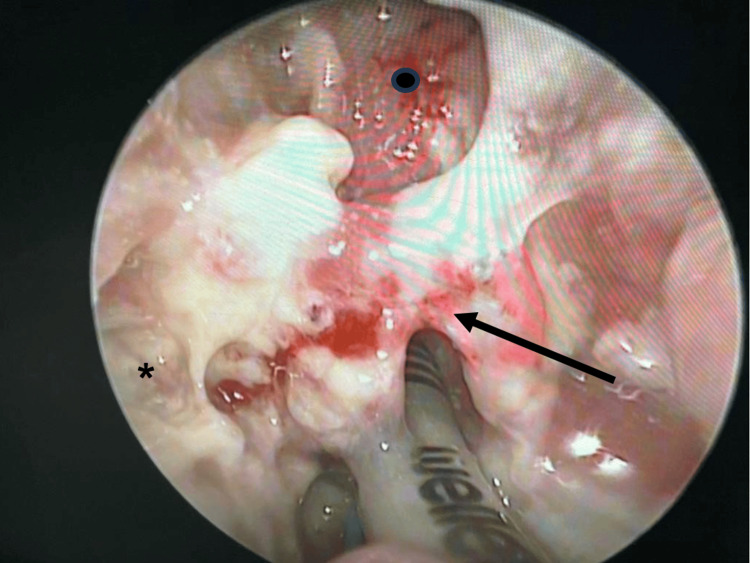
Slough is observed over the epiglottis (black arrow), piriform fossa (asterisk), and post-cricoid region (black dot). Copious secretions are also noted.

**Figure 5:**
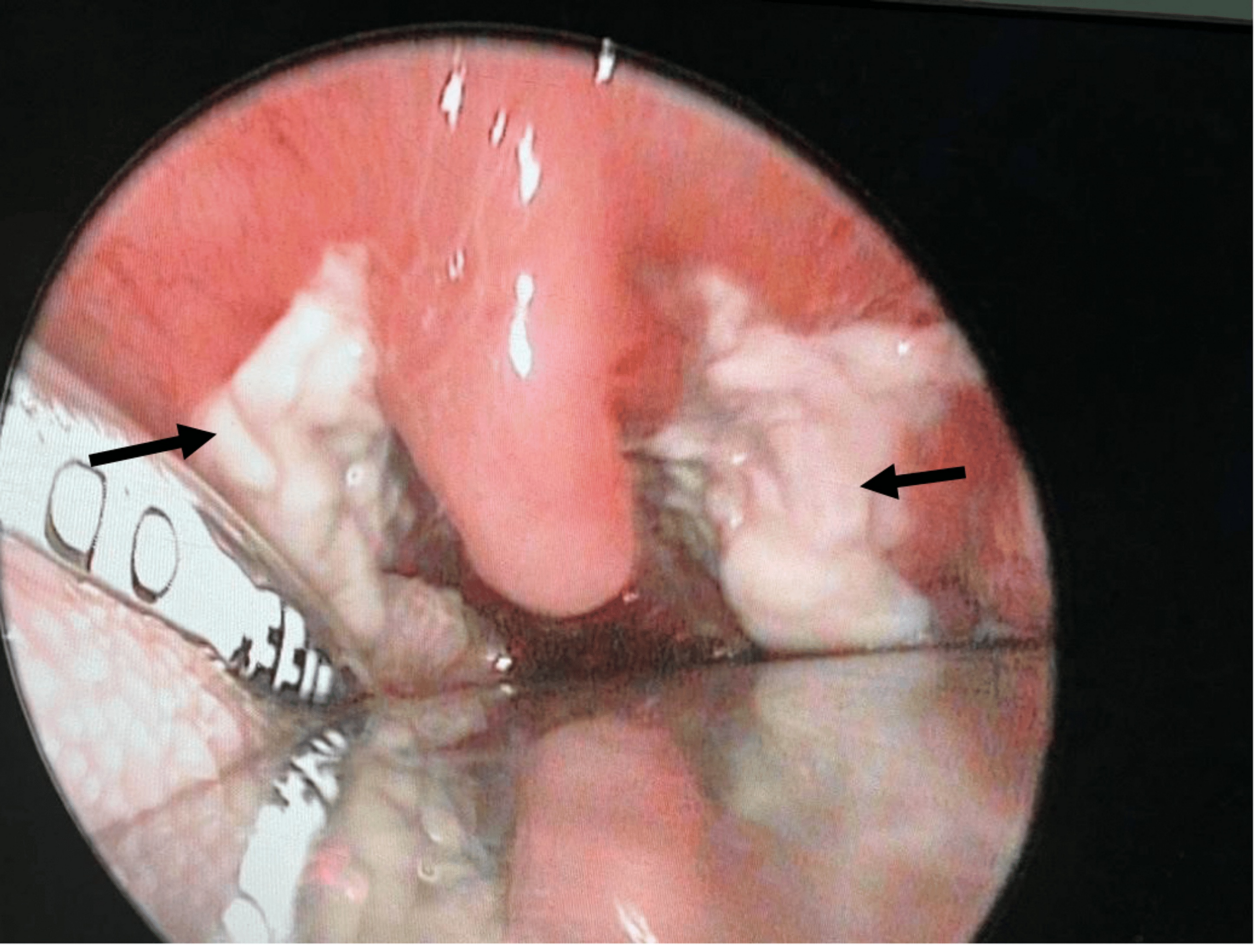
Thick slough is seen over the bilateral tonsils and edematous uvula. The black arrows indicate the bilateral tonsillar regions.

Examination under anesthesia (EUA) with direct laryngoscopy and nasoendoscopy was performed on the patient. Intraoperatively, a breach in the posterior pharyngeal wall mucosa at the level of the oropharynx was observed, suggesting a possible rupture of the collection. Slough was noted over the oropharynx, supraglottic region, arytenoids, piriform fossa, and post-cricoid area (Figure 4). The mucosa of the oropharynx and hypopharynx appeared friable. The epiglottis and bilateral false vocal cords were edematous. Slough was also present over the bilateral tonsils and the posterior pharyngeal wall (Figure 5). Aspiration of the bilateral tonsil and peritonsillar areas was performed, but no abscess was aspirated. Nasoendoscopic examination revealed slough on the left middle turbinate and right nasal septum, with adenoid hypertrophy but no slough in the nasopharynx. Biopsies taken from the bilateral tonsils, the laryngeal surface of the epiglottis, and the left aryepiglottic fold showed necrosis. Overall, the tissue biopsy indicated acute inflammation and abscess formation.

**Figure 6:**
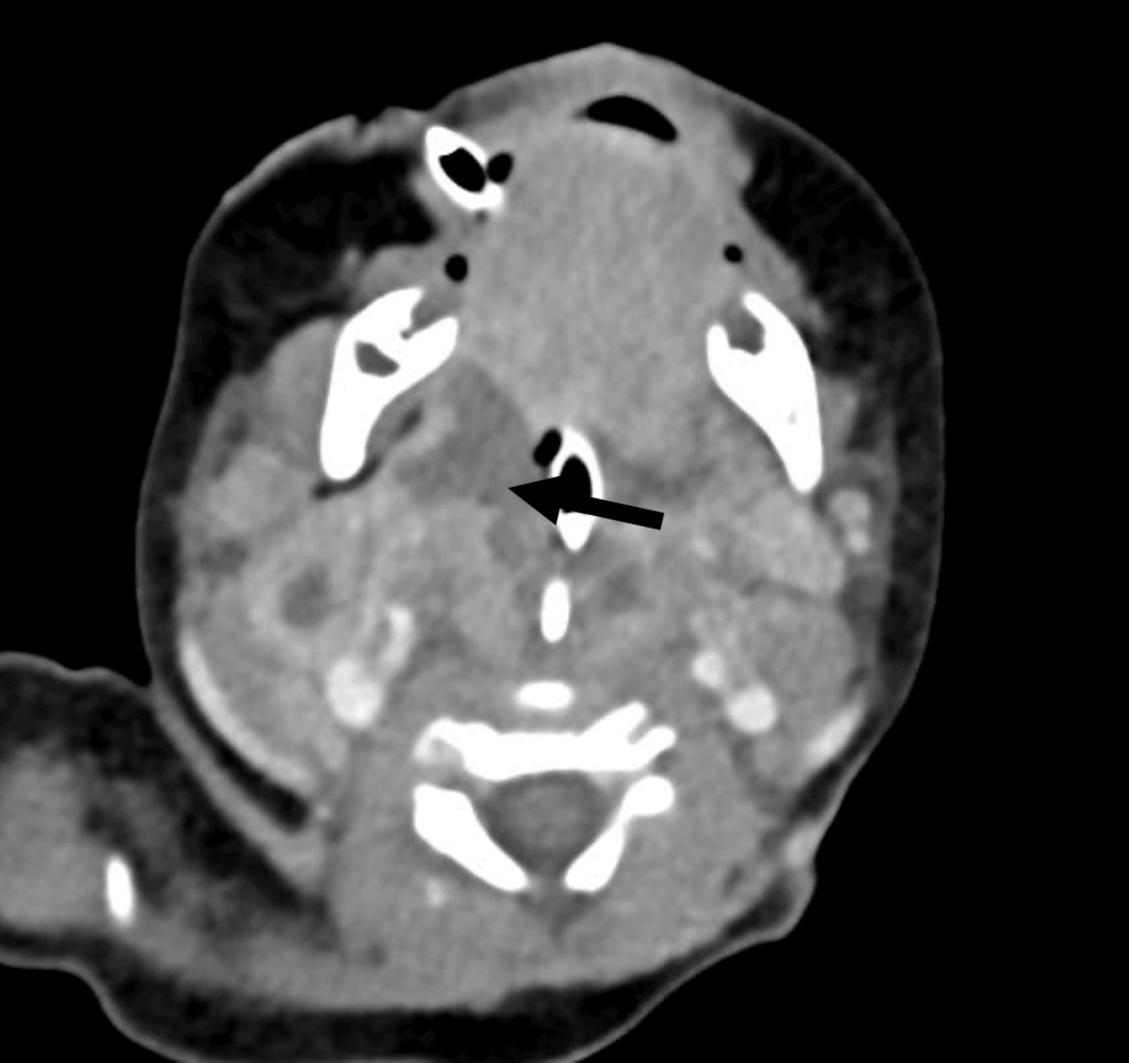
Laterally, the collection (black arrow) has extended toward and anterior to the bilateral carotid spaces in the post-styloid parapharyngeal fossa. Mild compression of the right IJV is noted posteriorly; however, the right IJV and right common carotid arteries remain patent with no filling defects observed. The borders of the collection are similar to those seen in the initial scan. Multiple enlarged, lobulated cervical lymph nodes at bilateral levels II and V show a new area of central hypodensity, suggestive of central necrosis. IJV, internal jugular vein

Both laryngeal tissue samples sent for culture and sensitivity testing grew *Pseudomonas aeruginosa*, which was sensitive to ceftazidime and gentamicin. Tracheal aspirate cultures also grew* P. aeruginosa* with sensitivity to ceftazidime, amikacin, gentamicin, and cefepime. Tissue cultures were negative for diphtheria. The patient initially received IV ceftriaxone BD for nine days, IV metronidazole TDS for five days, and IV gentamicin OD (7 mg/kg) for four days. Given the results of the pseudomonal cultures, the antibiotic regimen was escalated to IV cefepime BD (50 mg/kg/dose) for two weeks and IV amikacin BD (15 mg/kg in divided doses) for one week. A repeat CECT of the neck was subsequently performed, revealing enlarging tonsillar and peritonsillar multiloculated abscesses with worsening extension into the retropharyngeal space at the level of the nasopharynx (Figure 6).

**Figure 7:**
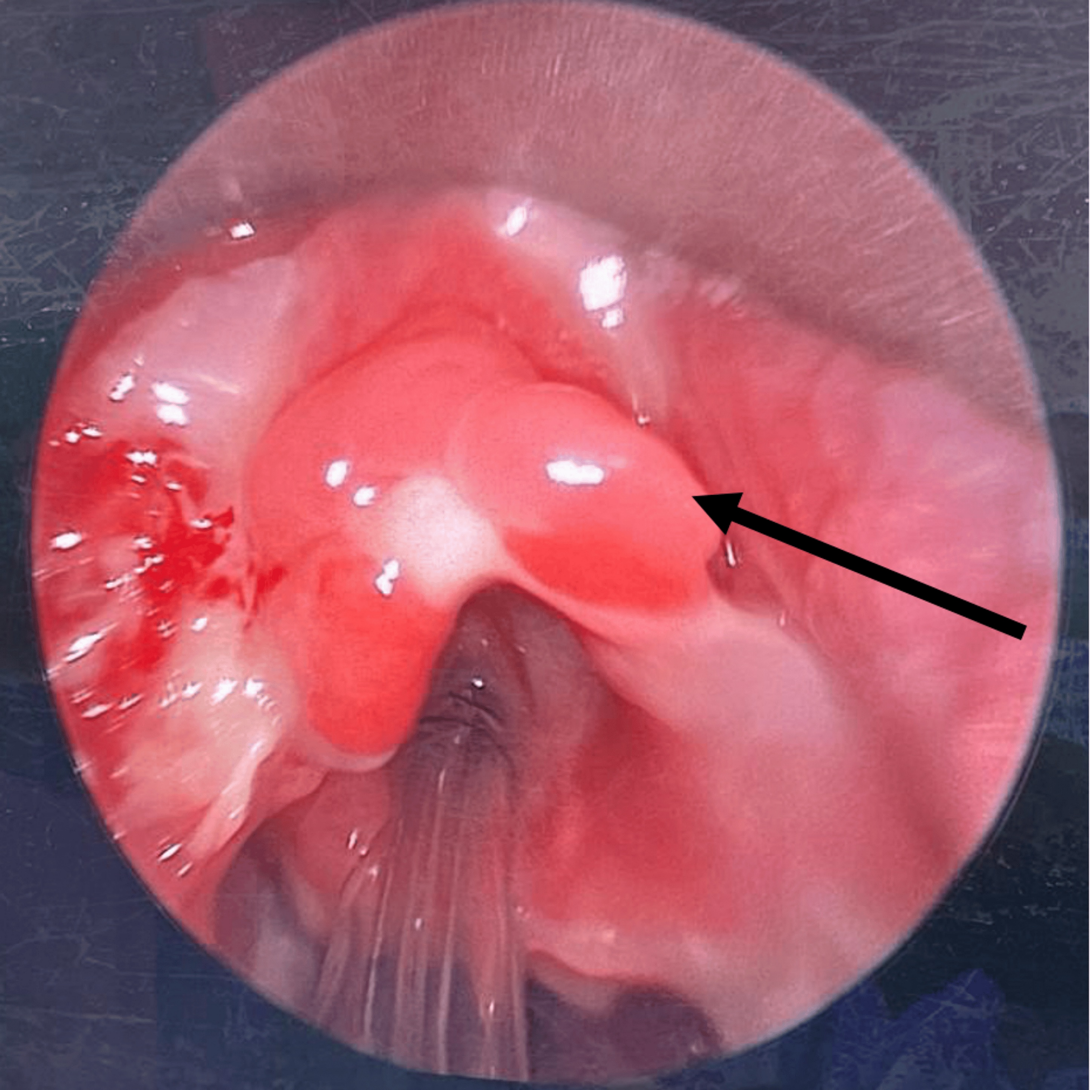
Reduction in slough is observed at the epiglottis and vallecula. The epiglottis appears less edematous (black arrow).

**Figure 8:**
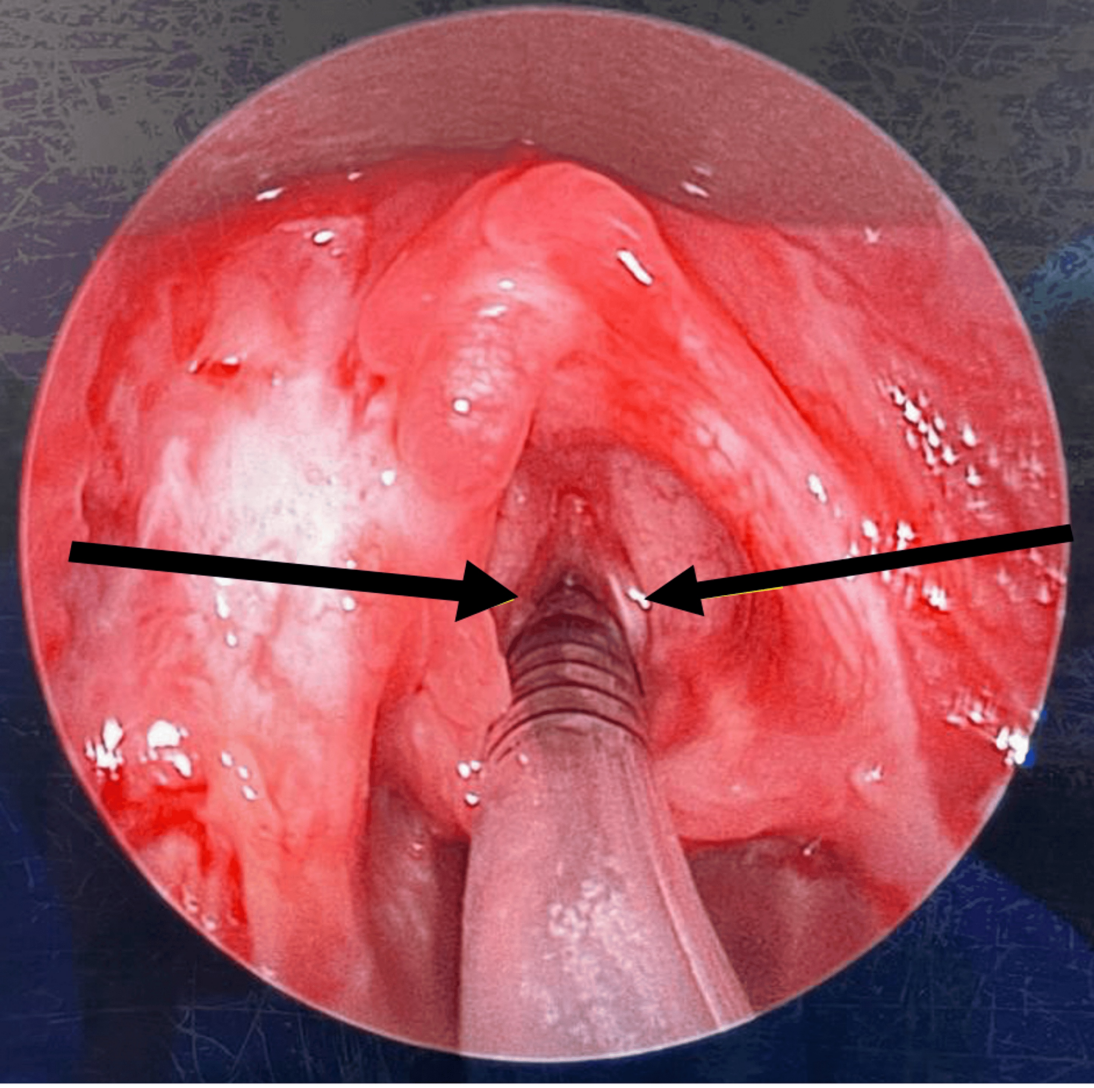
Normal bilateral vocal cords (black arrows).

The patient underwent a second direct laryngoscopy, EUA, and tracheoscopy after nine days. Intraoperatively, the bilateral tonsillar tissues remained sloughy and necrotic, extending to the tonsillar fossa. However, there was a reduction in the sloughing observed at the left vallecula, bilateral piriform fossa, and posterior pharyngeal wall. The epiglottis appeared less edematous (Figure 7). Aspiration of the bilateral intratonsillar and peritonsillar regions yielded no pus. Tracheoscopy revealed normal bilateral vocal cords and an unremarkable subglottic region and trachea up to the carina (Figure 8).

**Figure 9:**
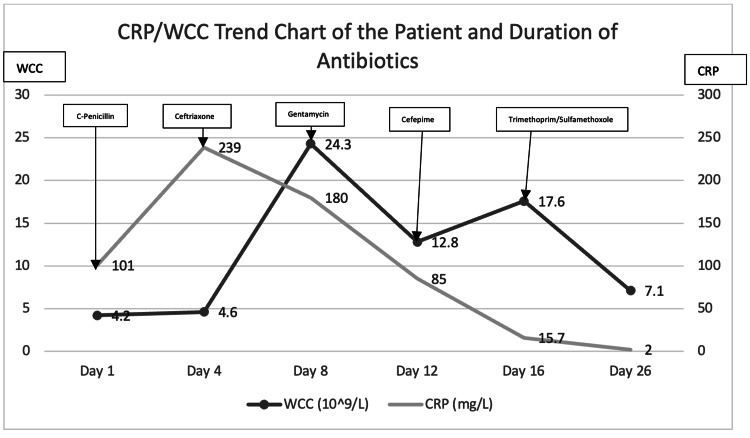
CRP/WCC trend chart of the patient and duration of antibiotics

Multiple biopsies taken from the left vallecula, bilateral tonsils, bilateral middle turbinates, and the left septal wall were sent for histopathological examination, which revealed necrotic tissue with an acute inflammatory process. Tissue cultures from the left vallecula and bilateral tonsils grew *P. aeruginosa *and* S. maltophili*a. The latter organism was multidrug resistant but sensitive to cotrimoxazole. Consequently, IV trimethoprim/sulfamethoxazole was initiated at a dose of 8 mg/kg/day in divided doses, administered twice daily for one week. The patient responded well to this treatment, showing signs of recovery and a decline in infective markers. Figure 9 summarizes the CRP/WCC trend in relation to the duration of the antibiotics administered.

After being intubated for 17 days, the patient was successfully extubated in a controlled setting and subsequently weaned off to room air. Prior to extubation, direct laryngoscopy revealed resolution of slough over the epiglottis, base of the tongue, vallecula, and posterior pharyngeal wall. Although some slough remained over the bilateral tonsils, desloughing was achieved. The patient was observed in the PICU for an additional three days before being transferred to the general pediatric ward and later discharged.

In total, the patient spent one month in the hospital but made a complete recovery, being discharged stable on room air and able to tolerate regular feeding. The final diagnosis was necrotizing tonsillitis with retropharyngeal abscess. He was discharged with oral trimethoprim/sulfamethoxazole three times a week (5 mg/kg) as prophylaxis for an additional three weeks. The patient continues to follow up regularly with ENT and pediatric outpatient clinics. Investigations for an immunocompromised state were normal. As of 15 months post-discharge, the patient has remained well.

## Discussion

Necrotizing tonsillitis is a rare but aggressive infection of the tonsils that poses a high risk of progressing to airway compromise. Although infrequent, cases have been documented with herpes simplex virus as a common pathogen [2,3]. Typical symptoms include fever, sore throat, lymphadenopathy, dyspnea, inflamed tonsils (with or without necrosis), and a history of ineffective antibiotic treatments. While most tonsillitis cases are caused by viruses like influenza A, parainfluenza, Epstein-Barr, and respiratory syncytial virus, which usually resolve with symptomatic treatment [4], tuberculosis (TB) can also be a causative agent in regions where TB is prevalent [5,6]. In this case, the infection was due to *P. aeruginosa *and superinfection by *S. maltophilia*, an opportunistic gram-negative bacillus that primarily affects immunocompromised individuals [7-10]. A retrospective review by Büyükcam et al. reported a mortality rate of 33.3% for this infection, although estimates vary between 6% and 40% [1]. The acute onset and rapid deterioration of the patient led to airway compromise secondary to necrotizing tonsillitis complicated by a retropharyngeal abscess.

Retropharyngeal abscess is a rare but potentially serious condition in infants, characterized by pus accumulation in the retropharyngeal space. Clinical signs include persistent high-grade fever, dysphagia, neck pain or stiffness, and stridor. Rapid abscess progression or significant swelling can lead to respiratory distress. Airway management is crucial in these cases; hence, elective intubation was necessary and could not be postponed while awaiting the effects of IV antibiotics.

In such scenarios, isolating the organism through culture and initiating targeted antibiotic therapy is essential. EUA during direct laryngoscopy and biopsy provided critical information on the extent and severity of the infection. The presence of breached posterior pharyngeal wall mucosa at the oropharyngeal level suggested a likely rupture of the retropharyngeal abscess. This, along with extensive sloughing and inflammation affecting various pharyngeal and laryngeal structures, confirmed a severe necrotizing process. Notably, sloughing was observed not only in the oropharynx but also in the supraglottic, arytenoid, piriform fossa, and post-cricoid regions, highlighting the extensive nature of the disease. Examination under general anesthesia in a controlled operating room setting is safer and more effective than flexible nasopharyngolaryngoscopy, which risks aspiration or laryngospasm.

A second direct laryngoscopy under anesthesia allowed reassessment of the oropharyngeal infection’s progress. A repeat CECT scan of the neck revealed enlarging tonsillar and peritonsillar multiloculated abscesses with worsening extension into the retropharyngeal space. This assessment provided an opportunity for incision, drainage, or removal of slough as needed. In such cases, it is prudent to repeat tissue cultures and sensitivities if the patient’s condition does not improve despite appropriate IV antibiotics based on initial cultures. In this instance, two out of three tissue samples grew *S. maltophilia*, which was sensitive to trimethoprim/sulfamethoxazole. The patient’s condition and septic parameters improved only after a week of IV trimethoprim/sulfamethoxazole treatment for the superinfection.

A final direct laryngoscopy was performed to evaluate the progression of airway edema, allowing us to monitor treatment response and plan for extubation. Intraoperatively, there was a significant reduction in slough and edema in the supraglottic structures. This clinical and biochemical improvement gave us the confidence to extubate the patient, who was closely monitored for 24 hours before being transferred to the ward.

## Conclusions

Necrotizing tonsillitis caused by multidrug-resistant organisms such as *P. aeruginosa *or *S. maltophilia* can lead to severe, life-threatening complications like upper airway obstruction. In these critical situations, effective airway management is crucial and involves early recognition, prompt intervention, and meticulous monitoring. It is essential to obtain relevant tissue and blood cultures along with antibiotic sensitivity tests to guide targeted therapy. EUA during direct laryngoscopy allows for the collection of tissue samples for culture and provides an opportunity to evaluate the severity and extent of airway edema. Surgical intervention, including incision and drainage, aspiration, and desloughing under general anesthesia, can significantly aid in the recovery process. Successful management of such complex cases requires a multidisciplinary approach, involving pediatricians, otolaryngologists, intensivists, radiologists, and infectious disease specialists, to ensure comprehensive care and optimal outcomes.

## References

[1] Büyükcam A, Bıçakcıgil A, Cengiz AB, Sancak B, Ceyhan M, Kara A (2020). Stenotrophomonas maltophilia bacteremia in children - a 10-year analysis. Arch Argent Pediatr.

[2] Borhan WM, Dababo MA, Thompson LD, Saleem M, Pashley N (2015). Acute necrotizing herpetic tonsillitis: a report of two cases. Head Neck Pathol.

[3] Hirzel C, Nueesch S, Wendland T, Langer R (2016). Necrotizing herpes-simplex virus tonsillitis mimicking peritonsillar abscess. Infection.

[4] Wang Q, Du J, Jie C, Ouyang H, Luo R, Li W (2017). Bacteriology and antibiotic sensitivity of tonsillar diseases in Chinese children. Eur Arch Otorhinolaryngol.

[5] Motahari SJ, Afshar P, Ghasemi M, Larijani LV, Sheidaei S (2019). Primary tonsillar tuberculosis: a forgotten clinical identity. Iran J Microbiol.

[6] (2020). World Health Organization global tuberculosis report. https://apps.who.int/iris/bitstream/handle/10665/336069/9789240013131eng.pdf.

[7] Jiang Z, Ren Y, Zhang C, Yin Y, Li C (2022). Community-acquired Stenotrophomonas maltophilia infection in a child: a case report and literature review. Infect Drug Resist.

[8] Rello J, Kalwaje Eshwara V, Lagunes L (2019). A global priority list of the TOp TEn resistant Microorganisms (TOTEM) study at intensive care: a prioritization exercise based on multi-criteria decision analysis. Eur J Clin Microbiol Infect Dis.

[9] Shah A, Singhal T (2021). Stenotrophomonas maltophilia as a cause of meningitis in an infant. Indian J Pediatr.

[10] Falagas ME, Kastoris AC, Vouloumanou EK, Dimopoulos G (2009). Community-acquired Stenotrophomonas maltophilia infections: a systematic review. Eur J Clin Microbiol Infect Dis.

